# Cardiovascular Involvement in Psoriasis, Diagnosing Subclinical Atherosclerosis, Effects of Biological and Non-Biological Therapy: A Literature Review

**DOI:** 10.7759/cureus.11173

**Published:** 2020-10-26

**Authors:** Sharathshiva Valaiyaduppu Subas, Vinayak Mishra, Vishal Busa, Ishan Antony, Suganya Marudhai, Mauli Patel, Ivan Cancarevic

**Affiliations:** 1 Internal Medicine, California Institute of Behavioral Neurosciences & Psychology, Fairfield, USA; 2 Internal Medicine, Grant Medical College, Mumbai, IND; 3 Internal Medicine, Royal College of Surgeons in Ireland (RCSI) School of Medicine, Dublin, IRL

**Keywords:** psoriasis, cardio vascular disease

## Abstract

Psoriasis is a long-lasting, noncontagious chronic inflammatory disease of skin and joints. Previous epidemiological studies have demonstrated that psoriatic patients have a shorter life expectancy, mainly due to cardiovascular (CV) events with a higher prevalence of cardiovascular risk factors like dyslipidemia, diabetes mellitus, insulin resistance, obesity, and hypertension. Besides these risk factors, psoriasis likely plays an independent role in increasing CV events probably due to the chronic inflammatory state. This literature review aims to summarize the mechanism of atherosclerosis formation, CV risk factors, tools to diagnose subclinical atherosclerosis, and the effects of various therapies in psoriatic patients to prevent cardiovascular-related deaths in psoriasis. This review was performed by searching the relevant articles in PubMed and Google Scholar databases without including any exclusion criteria and time limitations. Our review documented that psoriatic patients are at increased risk of CV events due to chronic inflammatory profile and the associated CV risk factors. Also, anti-inflammatory therapies may prevent early subclinical atherosclerotic vascular changes reducing cardiovascular events. However, the available studies lack to establish the exact targets for CV risk factors, to assess the clinical importance of screening for subclinical vascular changes and the impact of anti-inflammatory therapies on CV risk profile in psoriatic patients. This heightened awareness about the CV involvement in psoriasis should encourage conducting large, well planned comprehensive studies to address these issues that can reduce cardiovascular morbidity and mortality.

## Introduction and background

Cardiovascular disease (CVD) is the general term for the group of disorders of heart and blood vessels. As per the World Health Organization, approximately 17.9 million people died from CVDs in 2016, representing 31% of all global deaths [1]. Of these deaths, 85% are due to heart attack and stroke. CVD was responsible for 840,768 deaths (635,260 cardiac) in the year 2016 and remains the major cause of death in the United States [1]. The estimated annual total cost of CVD in the United States in 2014-2015 was $351.2 billion, with $213.8 billion in direct cost, including 46% for inpatient care. Coronary heart disease is the leading cause of death attributable to CVD, followed by stroke, high blood pressure, heart failure, and others [2]. Every 37 seconds one person dies because of the CVD in the United States [3].

Atherosclerosis is the most common pathological process that leads to CVD, by the formation of atherosclerotic plaques consisting of necrotic cores, calcified regions, accumulated modified lipids, inflamed smooth muscle cells, endothelial cells, leukocytes, and foam cells [4]. These features of atherosclerotic plaques illustrate that it is a complex disease, which involves the components of vascular, metabolic, and immune systems in the process. Even though low-density lipoprotein remains the most significant risk factor for atherosclerosis, immune and inflammatory mechanisms of atherosclerosis have gained enormous interest in the past 20 years [4-6]. The American Heart Association/American College of Cardiology guidelines of 2018 - 2019 recommends consideration of risk factors in borderline to intermediate-risk individuals for primary prevention of atherosclerotic cardiovascular disease. The risk factors include high-risk race or ethnicity, family history of premature atherosclerotic cardiovascular disease, chronic kidney disease, metabolic syndromes, and chronic inflammatory disorders like rheumatoid arthritis, psoriasis, etc. [7].

Psoriasis, the chronic inflammatory skin disease, affects 2-3% of the general population and the prevalence is influenced by geographic location, age, and genetics [8]. The elevated levels of unspecific inflammation markers, proinflammatory cytokines, and immune cells in the circulation of psoriasis patients as compared with healthy controls support that psoriasis is more than skin deep. The systemic manifestation of psoriasis results in comorbidities that include, but not limited to, metabolic syndrome, cardiovascular disease (CVD), diabetes, depression, and cancer [8].

Previous epidemiological studies showed that severe psoriasis was associated with a 57% increased risk of cardiovascular mortality, and the cardiovascular disease risk was particularly high in young patients with a severe form of the disease [9-11]. It has been proposed that the incidence of premature atherosclerosis related to psoriasis may be one of the underlying causes of increased cardiovascular disease risk. Moreover, some studies have suggested that systemic inflammation in psoriasis may affect the vascular endothelium, in addition to skin, as in other inflammatory diseases such as rheumatoid arthritis [11]. Likewise, over the years the evidence has shown that psoriasis is associated with CVD, which may lead to an increased cardiovascular event and death rate. These data are mostly from observational studies and meta-analyses and indicate a potential pathogenetic link between these two systemic diseases, however definite proof of this relationship awaits further prospective studies [12]. Also, few studies are showing no relation between CVD and psoriasis [13, 14]. Regardless of the growing number of studies showing the evidence for the association between psoriasis and CVD, there are still many unsolved questions. It includes dealing the role of psoriasis as an independent risk factor for cardiovascular (CV) events, the influence of psoriasis severity and duration on CV damage, the detection of reliable parameters (clinical, laboratory, and/or instrumental) to stratify CV risk, and the use of biological and non-biological agents to prevent CVD-related deaths in psoriatic patients.

In this literature review, we are trying to shed some light on the potential link between psoriasis and CV events, pathogenesis, parameters to stratify the CV risk, and the effects of biological and non-biological agents in CVD prevention. Also, we are trying to summarize the results and conclusions from previous studies about CVD and psoriasis by doing a literature search using the PubMed and Google Scholar database. This summary of evidence will provide updated information for current clinical practice and to support the need for heightened awareness and appropriate screening for cardiovascular disease in patients with psoriasis.

## Review

Pathophysiology behind cardiovascular disease in psoriasis

Epidemiologic studies have shown that psoriasis is linked with a higher incidence of cardiovascular events. Using the research data collected from 1987 to 2002, Gelfand et al. reported that the myocardial infarction (MI) incidence was 3.58, 4.04, and 5.13 per 1000 person-years for unaffected controls, mild psoriatic and severe psoriatic patients respectively, after controlling for classic cardiovascular risk factors [10]. This suggests a pathogenetic role for disease-specific risk factors since the classic cardiovascular risk factors alone do not explain the high incidence of cardiovascular mortality and morbidity that is associated with psoriasis [10, 15]. The previous data showed that immune dysregulation and chronic inflammation are involved in the development, progression, and complications of atherosclerosis [16]. Psoriasis is marked by the Th-1 and Th-17 polarization of the adaptive immune response, later followed by the increase in expression of inflammatory mediators like interferon gamma (IFN-γ), tumor necrosis factor alpha (TNF α), interleukin-2 (IL-2), and interleukin-17 (IL-17) [17]. These inflammatory mediators play an important role in psoriatic plaque development. They stimulate keratinocyte proliferation and angiogenesis and further promote metabolic abnormalities, insulin resistance, and endothelial dysfunction. This contributes to the formation and progression of atherosclerotic plaque [16, 17]. The cytokine IL-17 can exhibit either anti-atherogenic or pro-atherogenic effects, depending on the inflammatory conditions, hence its role in atherosclerosis is still controversial [18]. In atherosclerotic plaques, cells showing both IFN-γ and IL-17 are found. In the presence of anti-inflammatory factors the induction of IL-17 may have protective effects [18]. Pro-inflammatory cytokines activate serine kinases including IκB kinase complex β and c-Jun N-terminal kinase which in turn inhibit insulin receptor substrates-1 (IRS-1) [19]. The inhibition of IRS-1 leads to impairment of phosphoinositide-3-kinase dependent pathway with subsequent overstimulation of the mitogen-activated protein kinase pathway. This pathway-specific insulin receptor plays a key role in the pathogenesis of endothelial dysfunction by decreasing the production of nitric oxide and increasing the secretion of endothelin-1 [19]. Insulin resistance develops since the metabolic functions of insulin are mediated by phosphoinositide-3-kinase dependent pathway by Akt-dependent phosphorylation of downstream effectors that regulate glucose uptake, glycogen synthesis, and lipolysis. The exposure to pro-inflammatory cytokines can also lead to the expression of adhesion molecules on the cell surface [19]. In due course, the intensification of inflammation can increase the expression of matrix metalloproteinase causing degradation of the collagen of the fibrous cap, which may determine the destabilization and rupture of atherosclerotic plaques [20]. Hence chronic inflammation may lead to atherosclerosis formation in psoriatic disease, which increases the CV risk [21].

Diagnosing subclinical atherosclerosis and risk assessment

Coronary Artery Calcium

Coronary artery calcium (CAC) is a well-proven marker to diagnose subclinical coronary atherosclerosis, which is firmly associated with future cardiovascular events [22]. Ludwig et al. found a remarkable increase in the prevalence (59.4% vs. 28.1%, P = 0.015) and severity (CAC score according to Agatston 3.7 vs. 0.0, P = 0.019) of CAC in patients with psoriasis. This study, by doing multiple linear regression calculations, identified psoriasis as an independent risk factor for CAC utilizing multiple linear regression calculations [23].

Coronary Plaque Characterization

The increased cardiovascular risk observed in psoriatic patients would be partially accountable to an elevated subclinical coronary artery disease burden composed of non-calcified plaques with high-risk features [24]. Lerman et al. conducted a study where all the subjects underwent coronary computed tomography angiography. The psoriatic patients were found to have increased total coronary plaque burden (1.22 ± 0.31 vs. 1.04 ± 0.22, p = 0.001), non-calcified coronary plaque burden (1.18 ± 0.33 vs. 1.03 ± 0.21, p = 0.004) and high-risk plaque prevalence beyond traditional risk factors (OR = 6.0, 95% CI: 1.1-31.7; p = 0.03), compared to the healthy volunteers [24]. At a one-year follow-up, they also found that there is an improvement of non-calcified plaque burden in psoriatic patients associated with modulation of remote skin inflammation, which suggests that the control of remote inflammation may lead to a reduction in the risk of developing coronary artery disease [24].

Carotid Intima-Media Thickness and Flow-Mediated Vasodilation

Several studies have demonstrated that early vascular abnormalities in psoriatic patients are marked by increased intima-media thickness of the common carotid artery and impaired flow-mediated vasodilation of the brachial artery. Balci et al. compared subclinical atherosclerosis of the carotid and brachial arteries in 43 psoriatic patients without cardiovascular risk factors and 43 matched healthy controls [25]. In psoriatic patients, mean intima-media thickness values of the right (0.607 ± 0.144 mm vs. 0.532 ± 0.101 mm, p = 0.006), left (0.611 ± 0.157 mm vs. 0.521 ± 0.117 mm, p = 0.003) and averaged common (0.609 ± 0.146 mm vs. 0.526 ± 0.104 mm, p = 0.003) carotid arteries were remarkably higher. Also, the mean flow-mediated vasodilation was significantly lower compared to controls (13.36 ± 6.39 mm vs. 19.60 ± 11.23 mm, p = 0.002). This shows the relationship between psoriasis and subclinical vascular damage seemingly unrelated to traditional risk factors [25].

Oxidation Modified Lipids (OML) - Oxidized Low-Density Lipoprotein (oxLDL), Oxidized High-Density Lipoprotein (oxHDL), Oxidized Lipoprotein(a) (oxLp(a))

A study by Sorokin et al. had shown that the oxidation modified lipids were elevated in psoriatic patients and were associated with coronary plaque burden, especially non-calcified burden (NCB), by the disease severity [26]. The paraoxonase (PON)-1 activity was also increased in psoriasis suggesting the possible compensatory anti-oxidative effect. Psoriasis subjects were found to have increased oxLp(a), lipoprotein(a) (Lp(a)) and oxHDL (p < 0.05 for all) with remarkable association of oxLDL (β = 0.10, p = 0.020) and oxHDL (β = −0.11, p = 0.007) with NCB. Also, the expression of PON1 (kU/μl) activity was higher compared to the healthy volunteers (8.55 ± 3.21 vs. 6.24 ± 3.82, p = 0.01) [26]. The study also demonstrates that there is a reduction in oxHDL (U/ml) (203.79 ± 88.40 vs. 116.36 ± 85.03, p < 0.001) with a concomitant decrease in NCB at one year (1.04 ± 0.44 vs. 0.95 ± 0.32, p = 0.03) associated with the anti-inflammatory treatment of psoriasis. Collectively, the study demonstrates the usefulness of OMLs and its association with early subclinical atherosclerosis in coronary arteries [26].

Lesional CD11b+ and Serum Myeloperoxidase

The elevation of serum myeloperoxidase (MPO) from activated myeloid cells is associated with increased cardiovascular risk through lipid oxidation, induction of endothelial dysfunction, and release of IL-12 from macrophages [27]. In a cross-sectional study done by Cao et al. 100 psoriatic patients and 53 controls, matched on gender, body mass index, and age were included to assess levels of MPO in serum. They also underwent immunohistochemical staining from psoriatic skin lesions, uninvolved skin of psoriatic patients, and normal skin. After adjusting for the traditional cardiovascular risk factors on which the groups differed, it was found that the serum MPO level was elevated 2.5-fold (P < 0.001) in psoriatic patients. MPO was highly expressed in le­sional psoriatic skin and colocalized predominantly with CD45+ CD11b+ leukocytes [27]. The circulating levels of MPO were correlated with the CD11b+ cell density. The elevated serum levels of MPO may be due to the activation of lesional skin CD11b+ leukocytes that in turn generate MPO. Thus the activity of lesional CD11b+ leukocytes may be an alternative to Psoriasis Area and Severity Index (PASI) in measuring the disease burden that underlies the MPO biomarker for systemic inflammation related to cardiovascular disease [27].

GlycA

GlycA is an emerging inflammatory biomarker that predicted cardiovascular events in population-based studies. GlycA is found to be associated with the severity of psoriasis and subclinical CVD beyond the traditional cardiovascular risk [28]. Joshi et al. conducted a two-stage, cross-sectional study design: first cohort (122 psoriasis patients and 109 controls; total = 231) and second cohort (151 psoriasis patients and 30 controls; total = 181). The GlycA was measured in the subjects by nuclear magnetic resonance spectroscopy. GlycA was found to be elevated in psoriatic patients compared to controls (first cohort: 408.8 ± 75.4 vs. 289.4 ± 60.2, p < 0.0001, second cohort: 415.8 ± 63.2 vs. 346.2 ± 46, p < 0.0001) and showed a dose-response with the severity of the disease [28]. In stage two, GlycA was found to be associated with vascular inflammation (β = 0.36, p < 0.001) and coronary artery disease (CAD) (β = 0.29, p = 0.004), beyond the cardiovascular risk factors in psoriasis. In receiver operating characteristic (ROC) analysis, GlycA added value in predicting vascular inflammation (p = 0.01) and CAD (p < 0.01) [28]. Also, the initiation of anti-tumor necrosis factor therapy (n = 16) reduced the severity of psoriasis (p < 0.001), levels of GlycA (463.7 ± 92.5 vs. 370.1 ± 78.5; p < 0.001) and vascular inflammation (1.93 ± 0.36 vs. 1.76 ± 0.19; p < 0.001). In this study, it is also found that GlycA remained associated with vascular inflammation (β = 0.56, p < 0.001) after the treatment. These findings from the study support the possible use of GlycA in assessing the subclinical CVD risk in psoriasis [28].

Cholesterol Efflux Capacity

Cholesterol efflux capacity (CEC) was recently shown to predict future risk of cardiovascular events. Psoriasis can both increase cardiovascular risk and impairs CEC. Salahuddin et al. assessed the relationship between coronary plaque burden and CEC in 101 psoriatic patients [29]. Coronary plaque burden was assessed by quantitative coronary computed tomography angiography (CCTA) and CEC was quantified using a cell-based ex vivo assay measuring the ability of apoB depleted plasma to mobilize cholesterol from lipid-loaded macrophages. It was found that CEC was inversely proportional to noncalcified coronary plaque burden (unadjusted β-coefficient - 0.33; P < 0.001), and this relationship persisted after adjustment for cardiovascular risk factors (β-0.24; P < 0.001), high-density lipoprotein cholesterol levels (β-0.22; P < 0.001), and apolipoprotein A1 levels (β-0.19; P < 0.001) [29]. The result of this study shows that CEC is inversely associated with prevalent coronary plaque burden measured by quantitative CCTA. Hence, low CEC may be a significant marker for the diagnosis of subclinical coronary atherosclerosis in psoriatic patients [29].

Epicardial Fat Thickness (EFT)

The visceral adipose tissue around the heart affects the heart and coronaries by secreting pro-atherogenic mediators [30]. It can be evaluated easily by the measurement of EFT. In a study, 115 adult patients (62 male; mean age 33.6 ± 6.0 years) with psoriasis (Group 1) and 60 age- and sex-matched healthy individuals (28 male; mean age, 32.5 ± 8.3 years) (Group 2) were included to find the relationship between EFT and psoriasis. EFT was obtained by transthoracic echocardiography. EFT was found to be significantly higher in Group 1 than in Group 2 (5.7 ± 1.2 vs. 4.1 ± 1.0 mm, p < 0.001) [30]. The psoriasis disease activity score was found to be an independent predictor of EFT in patients with psoriasis (β = 0.21, t = 2.67, p = 0.01). The findings from this study show that psoriatic patients compared with the controls had significantly higher EFT [30].

Urinary Orosomucoid (u-ORM)

Recent studies have demonstrated that urinary orosomucoid (u-ORM) could be a more sensitive, non-invasive biomarker of inflammatory activation than serum orosomucoid. Orosomucoid or α-1-acid glycoprotein is another major acute-phase protein mainly produced by the liver [31]. In a study done by Németh et al. 114 psoriatic patients were enrolled. Urinary orosomucoid and urinary orosomucoid/urinary creatinine (u-ORM/u-CREAT) ratio showed a significant correlation with the QRISK score (u-ORM, r = 0.245; u-ORM/u-CREAT, r = 0.309) [32]. When comparing mild psoriatic patients to moderate psoriatic patients, significant differences could be found in the u-ORM and u-ORM/u-CREAT ratio. This study exhibits a connection between urinary ORM and cardiovascular risk. U-ORM and u-ORM/u-CREAT ratio could be used as an indicator of low-grade inflammation in mild and moderate psoriasis [32].

Cardiogoniometry

Cardiogoniometry (CGM) is a spatiotemporal electrocardiologic method that can be used as a cardiovascular diagnostic tool. Poorzand et al. conducted a study with 30 psoriatic patients and 30 age and sex-matched healthy individuals, without any history of CVD or traditional cardiovascular risk factors [33]. Electrocardiogram (EKG) and CGM were performed in these subjects. They noticed a remarkable difference between the psoriatic patients and the controls regarding myocardial ischemia score (-1.53 ± 2.63 vs. -0.46 ± 0.73, p = 0.037) [33]. There was a moderate correlation found between QT dispersion and the duration of the disease (r = 0.369, p = 0.022). Myocardial ischemia score, standard deviation of normal R-R interval and heart rate had positive correlation with PASI (r = 0.481; p = 0.004, r = 0.427; p = 0.009 and r = 0.427; p = 0.009, respectively) but not with duration of the disease. This study shows that the resting EKG and CGM abnormalities and their correlation with disease severity raise concerns about the need for future cardiovascular follow-ups of psoriatic patients, especially in those with higher disease severity [33].

Biological and non-biological therapies

Statins

In Russia, a pilot study was conducted in the year 2007, to demonstrate the efficacy of statin (simvastatin) in treating psoriasis [34]. Patients selected for the study were at least 18 years of age, with plaque psoriasis covering >10 percent of their bodies and the psoriasis area and severity index (PASI) score of at least 12. Seven patients were prescribed simvastatin 40 mg/d for eight weeks and were followed up in the fourth and eighth weeks. During the follow-up, the patients were assessed for the severity of the disease using the PASI and dermatology quality of life index (DLQI). At the end of eight weeks, they found a statistically significant decrease in the mean PASI score by 47.34 percent, and also the DLQI score started to decrease [34]. At the end of the study, two patients achieved a 50% PASI response and two patients achieved a 75% PASI response. Even though this study had limitations which include a less number of patients, uncontrolled, and failed to assess lipid levels, their study findings suggest that statins could have clinical utility in treating psoriasis. Although it is not well-known whether the treatment of hyperlipidemia with statins in psoriatic patients will decrease their risk of CAD or other atherosclerotic diseases, theoretically statins should benefit them as it is proven to decrease the risk of clinical manifestation of the atherosclerotic process [34].

Nonselective Immunomodulators

Cyclosporine may cause or worsen dyslipidemia and hypertension. Oral retinoids can also cause dyslipidemia [35]. A prospective cohort study with 10,539 psoriatic patients was done to demonstrate their effects. The study reported that treatment with retinoids (acitretin) or cyclosporine was associated with a remarkable increase in the levels of total cholesterol (OR = 1.51 and 1.34), after follow-up at eight weeks and 16 weeks respectively, compared to baseline [35]. Also, a rise in triglycerides (OR = 1.43) in patients treated with acitretin and a consistent blood pressure elevation (incidence of hypertension = 2.4%, OR = 3.31) in patients treated with cyclosporine was noticed. However, the study had one limitation, which was the lack of a control group [35].

Methotrexate, a folic acid analog, is an anti-inflammatory drug used widely in the long-term management of moderate to severe psoriasis. Methotrexate may also exert opposite effects on the atherosclerotic process. Long-term therapy with methotrexate may increase the levels of homocysteine and cause endothelial dysfunction that opposes the protective anti-inflammatory effect of the drug [36]. Prodanovich et al. conducted a retrospective study on 7615 psoriatic patients. This study shows that patients treated with methotrexate showed a 27% reduced risk of vascular disease compared to those who did not receive methotrexate, with a significantly higher risk reduction for patients prescribed a low cumulative dose of methotrexate compared with higher doses (RR = 0.50, 0.31-0.79) and for patients receiving folic acid in addition to methotrexate (RR = 0.56, 0.39-0.80) [37].

Selective Immunomodulators

The major coronary vascular events are associated with the plasma levels of TNF-α in patients with acute coronary syndromes, even after adjusting to the traditional cardiovascular risk factors [38]. In a study of psoriatic arthritis patients, the use of TNF-α inhibitors reduced the development of carotid atherosclerotic plaques. After the treatment for more than four years with TNF-α inhibitors, the incidence of carotid lesions in patients treated with TNF-α inhibitors was 15.8% and it was 40.4% in patients who received nonselective immunomodulators like cyclosporine, methotrexate, sulfasalazine, leflunomide, and disease-modifying antirheumatic drugs [39]. In a study conducted by Avgerinou et al., they reported that there is a significant improvement of flow-mediated vasodilation and decreased intercellular adhesion molecule (ICAM)-1 levels in 14 psoriatic patients after treating with adalimumab for 12 weeks [40]. There is one retrospective cohort study including 8845 psoriatic patients that compares the effects of TNF-α inhibitors, oral agents or phototherapy, and topical therapy on the incidence of myocardial infarction (MI). This study recorded that the incidence of MI in the TNF-α inhibitor group, oral/phototherapy group, and topical therapy group was 3.05, 3.85, and 6.73 per 1000 patient-years, respectively (p < 0.001) after a median follow-up of 4.3 years [41].

The cytokine IL-23 is a member of the IL-12 family comprising of the two subunits IL-12p40 and IL-23p19, which may participate in several chronic inflammatory diseases [42]. The antibodies that target both IL-12 and IL-23 are briakinumab and ustekinumab. Briakinumab showed very promising results at first but it was withdrawn from the market because of frequent major adverse cardiovascular events (MACE) during phase one and phase two clinical trials [43]. In a meta-analysis of 22 randomized controlled trials, which compared the psoriatic patients treated with TNF-α inhibitors, IL-12/23 inhibitors, and placebo. This study reported only one MACE in the TNF-α inhibitors group (n = 1474) and one in the placebo group (n = 1812), but 10 MACE were reported in the group treated with IL-12/23 inhibitors (n = 3179) [44].

The pro-atherogenic mechanism of IL-17 may be due to increased recruitment and activation of the myeloid cells in the intima that results in an increased release of pro-inflammatory cytokines [45]. But few studies have also shown the protective effects of IL-17A [46, 47]. The protective effects can be attributed to decreased endothelial expression of vascular cell adhesion molecule-1. Also, increased levels of IL-17 can stimulate collagen synthesis by vascular smooth muscles contributing to the stability of the plaque [46, 47]. In a study conducted to evaluate the effects of treating psoriatic patients with anti-IL-17A antibody (secukinumab), during the 52-week monitoring period one CV event was reported in the placebo group (n = 202) whereas 15 arterial atherothrombotic events and one cardiovascular death were reported in the anti-IL-17A antibody-treated group (n = 587) [48].

## Conclusions

In conclusion, based on our current review, it is evident that individuals with psoriasis have an increased risk of cardiovascular events independent of the effect of traditional risk factors. This increased risk of cardiovascular events suggests that psoriasis alone is an independent risk factor for developing cardiovascular disease (CVD). Even though this relationship is demonstrated in numerous studies, the complexity associated with this relationship still needs to be explored in depth. In this review, we also tried to summarize the various diagnostic methods to assess the risk of subclinical CVD and the effects of various therapies to decrease cardiovascular events in psoriatic patients. Important issues that need further exploration include elucidating accurate pathogenetic mechanisms, identification of appropriate screening strategies for diagnosing subclinical CVD, effects of biological and non-biological treatments that can result in slower progression of subclinical CVD and reduction of clinical events among psoriatic patients. To address these crucial issues and to prevent cardiovascular-related morbidity and mortality in psoriatic patients, well planned prospective studies with larger samples and longer durations are needed.
